# Intense Pulsed-Light Therapy for Proliferative Haemangiomas of Infancy

**DOI:** 10.1155/2011/253607

**Published:** 2011-12-27

**Authors:** Marie Caucanas, Philippe Paquet, Frédérique Henry, Claudine Piérard-Franchimont, Marie-Annick Reginster, Gérald E. Piérard

**Affiliations:** Department of Dermatopathology, University Hospital of Liège, 4000 Liège, Belgium

## Abstract

Infantile haemangioma therapy has long been a wait-and-see policy. Since recent development of laser and light therapy, pulsed dye laser has been successfully used for treating superficial haemangiomas. Few studies have been published about treatment with intense pulsed light (IPL) to assess the risk/benefit of IPL in the treatment of infantile haemangiomas during their early proliferative phase. In the present retrospective cohort study, we retrieved data about a series of 14 Caucasian children (median age: 4.8 months) with infantile haemangiomas treated with Photoderm Vasculight flash lamp. All patients experienced a rapid regression of the haemangiomas after 3 treatments on average. Few adverse events were noted, including ulceration and crusts. No residual scarring and cosmetic damages were noticed. Fast growing haemangiomas should be treated with light therapy as soon as possible. This technology is safe, efficient, inducing regression, and preventing any further functional and aesthetic complications. The benefit-risk ratio favours the treatment of most types of haemangiomas which are out of the scope of betablocker administration.

## 1. Introduction

Haemangiomas are vascular tumours generally arising during the first weeks of life. They affect one in 10 infants, representing the most common vascular tumours occurring in infancy [1, 2]. Epidemiology data bring to light a higher prevalence rate in Caucasian newborns (1.1–2.6%) compared to Black and Japanese newborns (0.8%). Other risk factors include the female gender and prematurity with a body weight lower than 1500 grams at birth [2]. Little is known about the haemangioma physiopathology. It likely results from angiogenesis deregulation, but other contributing factors are suspected [3, 4]. The proliferative phase occurring during the first sixth months of life is usually followed by a stabilisation period leading to progressive regression generally completed before puberty [2, 5].

Pulsed dye laser (PDL) and intense pulsed light (IPL) are considered to be effective choices for a series of skin disorders including superficial haemangiomas [5–10]. IPL flash lamps emit broadband polychromatic high-intensity light in the wavelength spectrum ranging 515–1200 nm, and they target vessels at various depths inside the skin. The effect relies on selective photothermolysis, and the indication spectrum includes vascular tumours, pigmentary disorders, and excessive hair growth. Convertible cutoff filters allow treating a wide variety of skin conditions in a range of patient phototypes. In the treatment of haemangiomas, photons are absorbed by the endogenous chromophore haemoglobin of red blood cells, where the transfer of energy generates heat and leads to selective photothermolysis. Varied sizes and depths of vessels are possibly targeted. A major advantage is the large-size spot thanks to the square pulse design of the quartz inserted in the hand piece, allowing a complete treatment with only a few shots compared to the acuminate wave of lasers [5, 11].

IPL has notably been used in a study of 62 patients with infantile haemangiomas who received four to five IPL treatments at 4-week intervals with a clearance rate of more than 80% [5]. IPL with optimal pulse technology was considered as a safe and effective treatment modality for haemangiomas.

The aim of the present retrospective study was to assess the risks and benefits of IPL used in the early growth phase of proliferative haemangiomas of infancy.

## 2. Patients and Methods

The study design was approved by the University Hospital Research Review board. The parents received a complete information about the treatment procedure, and they signed an informed consent form.

### 2.1. Patients

**Figure 1:**
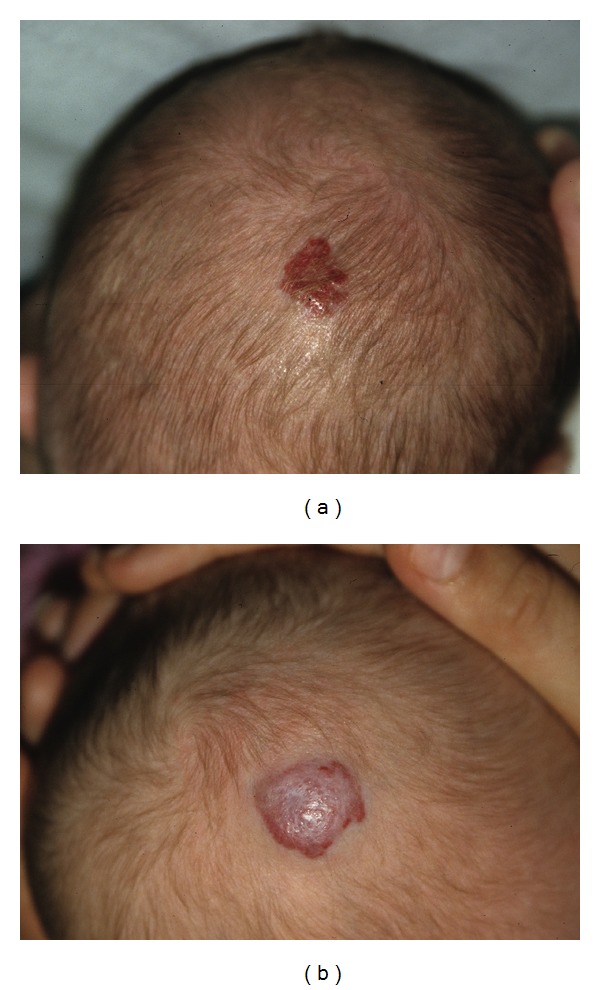
Infant 1 with haemangioma of the scalp, before and after 3 treatments with Photoderm.

**Figure 2:**
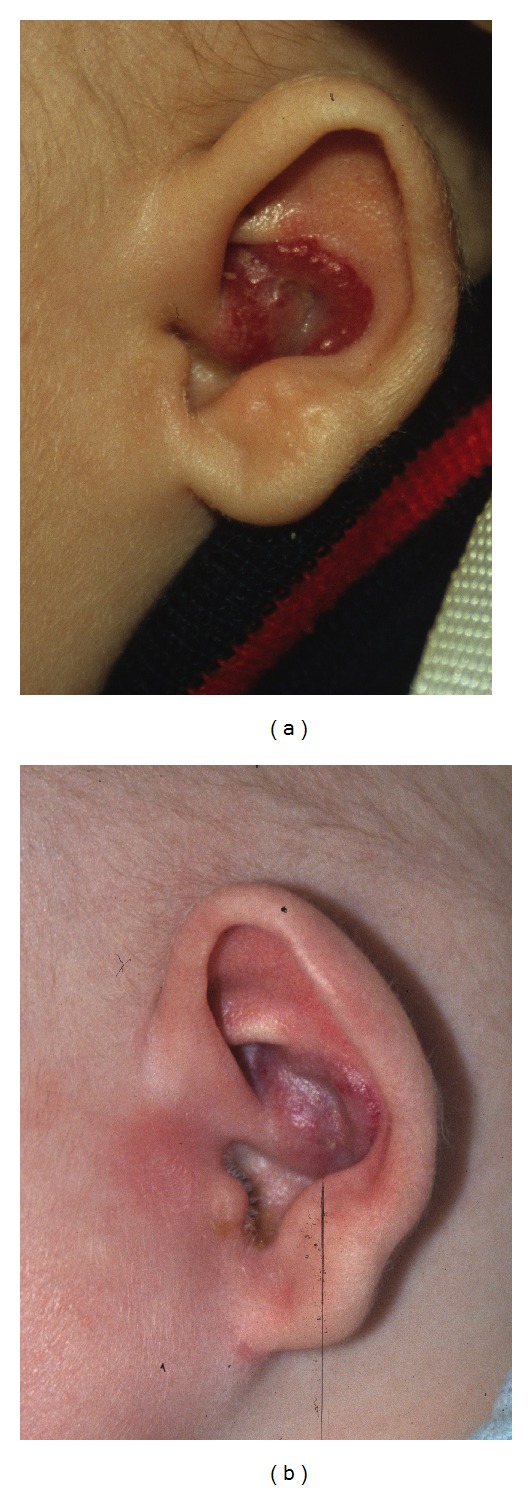
Infant 9 with haemangioma of the ear meatus, before and after 1 treatment with Photoderm.

**Figure 3:**
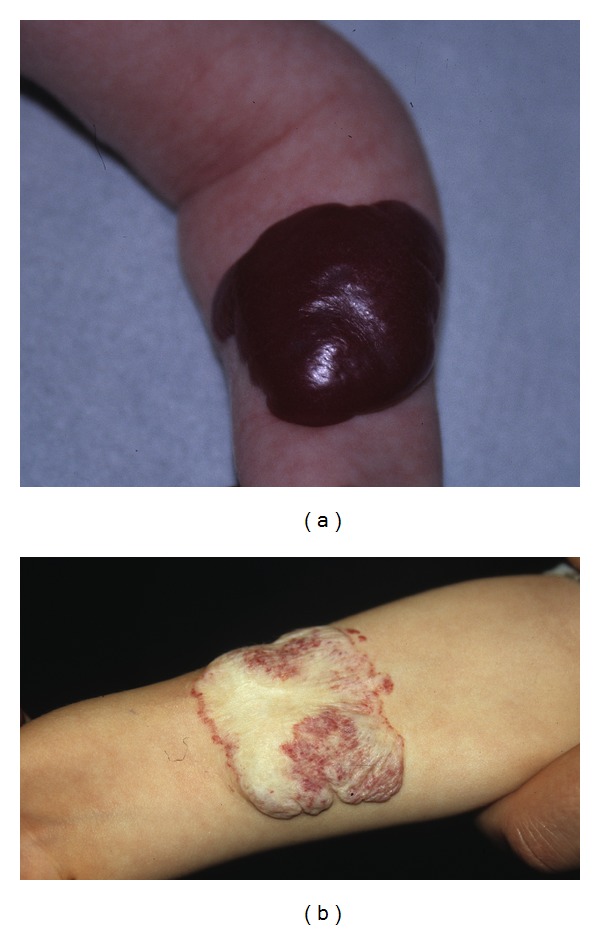
Infant 8 with haemangioma of the forearm before and after 3 treatments with Photoderm.

**Figure 4:**
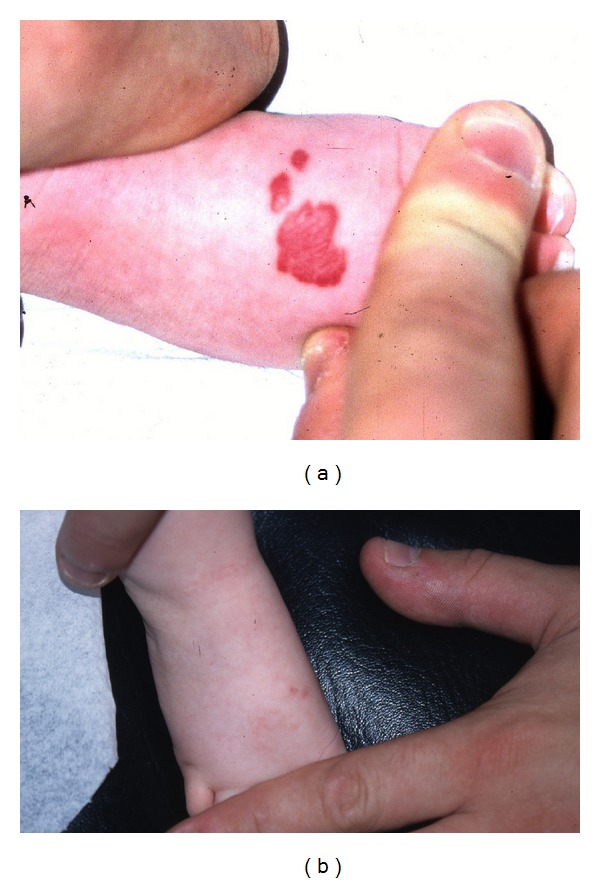
Infant 1 with haemangioma of the foot before and after 4 treatments with Photoderm.

A total of 14 infants (9 girls and 5 boys) with one or several infantile haemangioma(s) treated in the last five years (2007–2011) were included in this retrospective study. They had fair skin and a median age of 4.8 months. All but one were younger than 8 months (Table 1). The haemangiomas were variably located on the cheeks, eyebrows, nose, forehead, chin, neck, scalp (Figure 1), external ear meatus (Figure 2), back, thigh, forearms (Figure 3), feet (Figure 4), and hands. The most frequent locations were the head and neck. When measured and recorded, the average diameter of the haemangiomas reached about 2.5 cm (Table 1).

 The lesions were treated with the IPL Photoderm device. They were enrolled at a time when the effect of propranolol on infantile haemangiomas was not firmly established. The patients were selected when their larger diameter exceeded 1 cm, or when smaller lesions expanded 0.5 cm in diameter over 6–8 months. The haemangioma thickness was always less than 0.5 cm above the skin surface. The goal of the treatment was to halt the haemangioma growth. There was no patient with associated malformative syndrome. All but one had never received any previous treatment. One infant had indeed received a single intralesional corticosteroid injection. One patient was treated with IPL for potential impaired functional hearing complications (obstruction of the ear canal). The other 13 patients were treated to limit any cosmetic deformity outcome.

### 2.2. Treatment Parameters

The Photoderm flash lamp was used with 550 and 590 nm cutoff filters. Fluences ranged from 26 to 51 J/cm². Though the procedure is painful, no local anaesthesia was performed, considering that the shots only last a few milliseconds and babies stop crying right after the treatment.

### 2.3. Assessment

Photographs were taken at each visit, and tracings of the haemangioma margins were performed on transparent plastic sheets. The clinical observations were retrieved from the medical files.

## 3. Results

**Figure 5:**
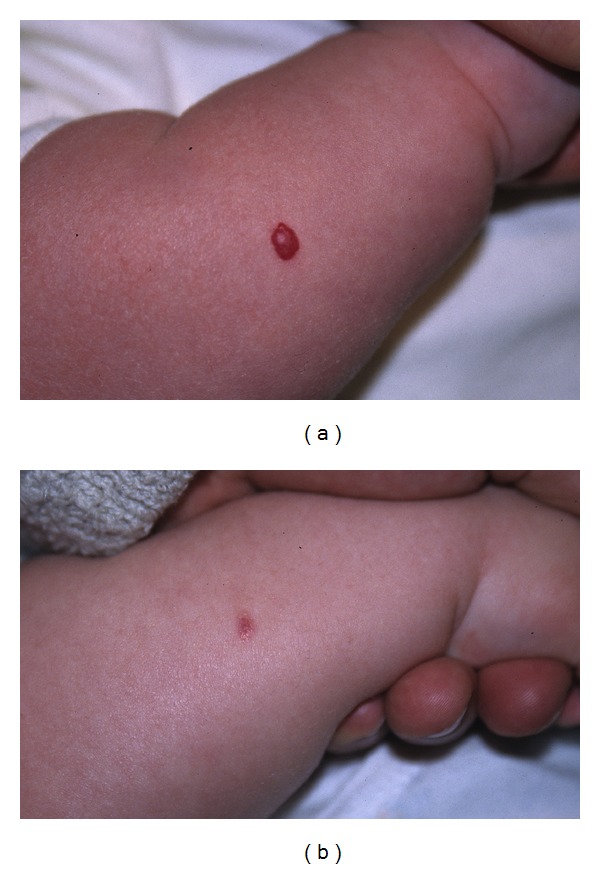
Infant 6 with haemangioma of the forearm, before and after 3 treatments with Photoderm.

Data are summarized in Table 1 and in Figures 1, 2, 3, 4, and 5. At each treatment session, one to 15 shots were delivered according to the size of the haemangioma. One to 6 treatments (median: 3) were performed with intervals ranging 1–12 weeks according to the clinical evolution. One month after the last treatment, all lesions were cleared, at least the exophytic part of the haemangiomas.

Few adverse events were recorded except bleeding, ulceration, and crusting after the first two treatment sessions in patient 5. By the end of the followup ranging 3–66 months (median: 10.5 months), no residual scarring and pigmentation were observed in any of the infants. No recurrences were experienced.

## 4. Discussion

In general, the speedy growth of haemangiomas during the first weeks of life reaches a maximum rate after three to six months, before entering a quiescent phase followed by a slow involution taking place between the 12th and 18th month of life [2]. The nonintervention policy remains an axiom, since solitary uncomplicated haemangiomas have a natural course to complete regression in 50% of the patients by the age of 5 years, and an additional 10% regression of lesions is reached per subsequent year [2, 12]. However, the actual evolution is unpredictable on the early proliferative phase, considering that some haemangiomas barely proliferate, whereas others reach a large size. Children bearing voluminous haemangiomas may suffer from a functionally and socially disabling condition. As haemangiomas may take more than ten years to complete involution, children are likely to be subject to teasing and shame, particularly at school where stigmatization most frequently occurs, leading to social withdrawal, anger, or aggressivity [13, 14].

After regression, voluminous infantile haemangiomas may leave a substantial residual cosmetic deformity with epidermal atrophy, persistent telangiectasias, fibrofatty or anetoderma-like saggy skin, or hypopigmentation [13–16]. More specific areas such as the nose, perioral skin, nasal side-wall, medial cheeks, and ear are more likely to heal with a residual scar. Scalp haemangiomas may result in significant residual alopecia [14, 16].

 Since most of the haemangiomas undergo complete spontaneous regression during childhood, a “watchful waiting” standpoint was generally recommended in paediatric, dermatology, and plastic surgery [2]. However, an early therapeutic intervention should be considered during the proliferating phase of rapidly growing infantile haemangiomas.

As the evolution of infantile haemangiomas is unpredictable, the present study supports early treatment of any uncomplicated haemangioma larger than 1 cm in diameter at the first visit or those rapidly growing more than 0.5 cm over the 6–8 first months of life in order to avoid further physical and psychological sequelae during childhood. In the present study, all but one infant were younger than 8 months at inclusion, corresponding to the average age limit for the growing stage of haemangiomas.

 Local treatments of uncomplicated haemangiomas include intralesional corticosteroids or bleomycin, topical imiquimod, cryotherapy, surgery, and electrosurgery [13, 14, 17]. Such treatments are frequently associated with scarring [5]. In addition, light therapy (PDL, Nd-Yag laser, and IPL) have emerged as effective treatments despite occasional dyschromia and scarring [5].

 PDL emits a monochromatic light which wavelength corresponds to the absorption peak of haemoglobin, leading to selective photothermolysis of cutaneous blood vessels [5–9]. Yet, its short wavelength (585–595 nm) limits the treatment to superficial lesions (1.2 mm in depth). IPL flash lamps generate pulsed or polychromatic high intensity light exhibiting a deeper cutaneous penetration because of the emitted light spectrum ranging 500–1300 nm, including typical haemoglobin-absorbed wavelengths. Convertible cutoff filters allow targeting varied sizes and depths of blood vessels and may be chosen according to the patient's skin type. In contrast with lasers, flash lamps have a larger spot size, allowing complete treatment with only a few shots [5, 11]. IPL with optimal pulse technology could therefore be regarded as a safe and effective modality for the treatment of haemangiomas [5]. Lately, the demonstration of propranolol efficacy in the management of haemangiomas led to a progress in the treatment of the most severe ones. However, its use is limited by the need of hospitalisation for close monitoring in order to control the risk of adverse effects such as hypoglycaemia, hypotension, and bradycardia [14, 16, 17]. We believe that propranolol remains a second-line treatment for aesthetic and functional purposes, except for periorificial regions.

 In this retrospective study, treating infantile superficial uncomplicated haemangiomas at their early stage of development with IPL appeared to stop their rapid growth and to initiate involution in 3 to 4 treatment sessions on average. This evolution prevented any unpredictable disfiguring and functionally impairment. In addition, it boosted the regression phase before schooling, thus avoiding harmful psychological consequences and residual cosmetic deformities. The study limitations include small sample size, impossible assessment of natural haemangioma evolution, absence of a control group of patients, very variable time of followup, and absence of comparison with PDL treated patients.

 In conclusion, IPL appears to be a safe and effective treatment for infants with superficial rapidly growing haemangiomas or haemangiomas larger than 1 cm. Contrasting with the “wait and see” standpoint, we advocate a rapid management of these vascular tumours. This implies an early referral to a dedicated dermatological light/laser centre, especially in collaboration with paediatricians and general practitioners with special interest.

## Figures and Tables

**Table 1:** Salient clinical characteristics of the 14 infants.

Patient	Gender	Age (month)	Localisation	Size (cm)	FR	Nb T	I (week)	FU (month)	AE	Cutoff F	Fl	Nb P	Add T
1	F	3.5	Scalp and foot	1.5	N	4	2	10	Cr	550–590	30–51	2–6	N
2	F	3.5	Neck	5	P, Bl	3	2–4	10	N	550	26–30	10–15	Oral CS
3	F	3	Cheek	NS	N	4	2-3	30	N	590	42	3	N
4	F	7.5	Chin	2.5	N	5	4–16	24	N	590	26–42	2–6	N
5	F	7.5	Back	NS	N	4	3-4	66	Bl, Ulc	590	30–51	4–8	N
6	M	1.7	Thigh and forearm	NS	N	3	1-2	2.3	Cr	590	46	10–14	N
7	F	2.8	Forehead	0.8	N	1		3	N	590	45	1	N
8	M	5	Cheek, forearm, back, and scalp	NS	N	3	3	11	N	590	45–48	3–5	N
9	M	2.5	Ear meatus	3.5	N	1		5.6	N	590	44	4	N
10	F	1.4	Scalp	1.5	N	3	2–12	3.5	N	590	45	3	N
11	F	8	Eyebrow	NS	N	4	4–8	16	N	550	26–38	2	N
12	M	5	Nose	NS	Bl	1		5	N	550	28	3	N
13	F	3.5	NS	NS	N	6	4–8	10.5	N	550–590	36–51	1	N
14	M	12	Back and hand	NS	N	2	52	24	N	550	26–32	1–3	N

Age = at first visit; FR = functional repercussion (at the time patient was seen)

Nb T, I = number of treatments, interval; FU = followup (= last seen at the age mentioned); AE = adverse events

cutoff F, Fl, Nb P = cutoff filters (nm), fluence (J/cm²), number of pulses per treatment; Add T = additional treatment

F = female; M = male; N = none; NS = not specified, C = clearance

P = pain; Bl = bleeding; Ulc = ulceration; Tel = telangiectasias; Cr = crusts

CS = corticosteroids.
